# Modular Synthesis and Patterning of High-Stiffness Networks by Postpolymerization Functionalization with Iron–Catechol Complexes

**DOI:** 10.1021/acs.macromol.2c02561

**Published:** 2023-03-15

**Authors:** Declan P. Shannon, Joshua D. Moon, Christopher W. Barney, Nairiti J. Sinha, Kai-Chieh Yang, Seamus D. Jones, Ronnie V. Garcia, Matthew E. Helgeson, Rachel A. Segalman, Megan T. Valentine, Craig J. Hawker

**Affiliations:** † Materials Department, University of California Santa Barbara, Santa Barbara, California 93106-5050, United States; ‡ Department of Chemical Engineering, University of California, Santa Barbara, Santa Barbara, California 93106-5080, United States; § Department of Mechanical Engineering, University of California, Santa Barbara, Santa Barbara, California 93106-5070, United States; ∥ Department of Chemistry & Biochemistry, University of California Santa Barbara, Santa Barbara, California 93106-9510, United States; ⊥ Materials Research Laboratory, University of California Santa Barbara, Santa Barbara, California 93106-5121, United States

## Abstract

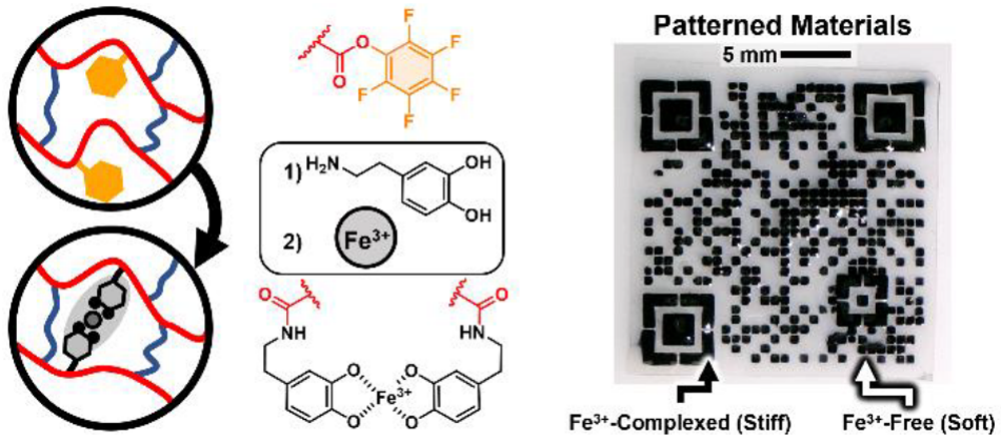

Bioinspired iron–catechol cross-links have shown remarkable success in increasing the mechanical properties of polymer networks, in part due to clustering of Fe^3+^–catechol domains which act as secondary network reinforcing sites. We report a versatile synthetic procedure to prepare modular PEG-acrylate networks with independently tunable covalent bis(acrylate) and supramolecular Fe^3+^–catechol cross-linking. Initial control of network structure is achieved through radical polymerization and cross-linking, followed by postpolymerization incorporation of catechol units via quantitative active ester chemistry and subsequent complexation with iron salts. By tuning the ratio of each building block, dual cross-linked networks reinforced by clustered iron–catechol domains are prepared and exhibit a wide range of properties (Young’s moduli up to ∼245 MPa), well beyond the values achieved through purely covalent cross-linking. This stepwise approach to mixed covalent and metal–ligand cross-linked networks also permits local patterning of PEG-based films through masking techniques forming distinct hard, soft, and gradient regions.

## Introduction

Structural biopolymers are characterized by outstanding performance and physical properties yet are derived from simple building blocks and self-assembling network motifs.^1−4^ In addition to covalent cross-linking, enhanced performance can arise from metal–ligand interactions and mineralization as in marine invertebrates,^5−9^ or through fiber reinforced systems in wood,^10^ cartilage,^11^ tendon,^12^ and adaptive soft tissues.^13^ These structural biopolymers rely on hierarchical network control through both localization of self-assembling motifs and chemical control of covalent and noncovalent cross-linking.^2−5,7^ This hierarchical control allows biological systems to achieve an extensive range of materials properties while also providing a set of design rules for the preparation of synthetic networks with enhanced performance.^2^

Synthetic network design has sought to mimic the high stiffness and toughness of biological materials through classical strategies such as modulating cross-link density,^14,15^ incorporating rigid fillers into soft polymer matrices,^16^ or tuning network crystallinity^17^ to alter network mechanical properties. More recent approaches have directly used bioinspired molecular motifs, allowing for the design of complex materials with hierarchical strengthening motifs. Examples include double networks,^18,19^ host–guest interactions,^20,21^ hydrogen bonding,^22^ and metal–ligand interactions,^23,24^ with metal–ligand systems having the added advantage of tunability via the strength and nature of the metal–ligand bond. This feature allows network stiffness and relaxation properties to be controlled through careful selection of metal–ligand dissociation times.^23,25−30^

**Figure 1:**
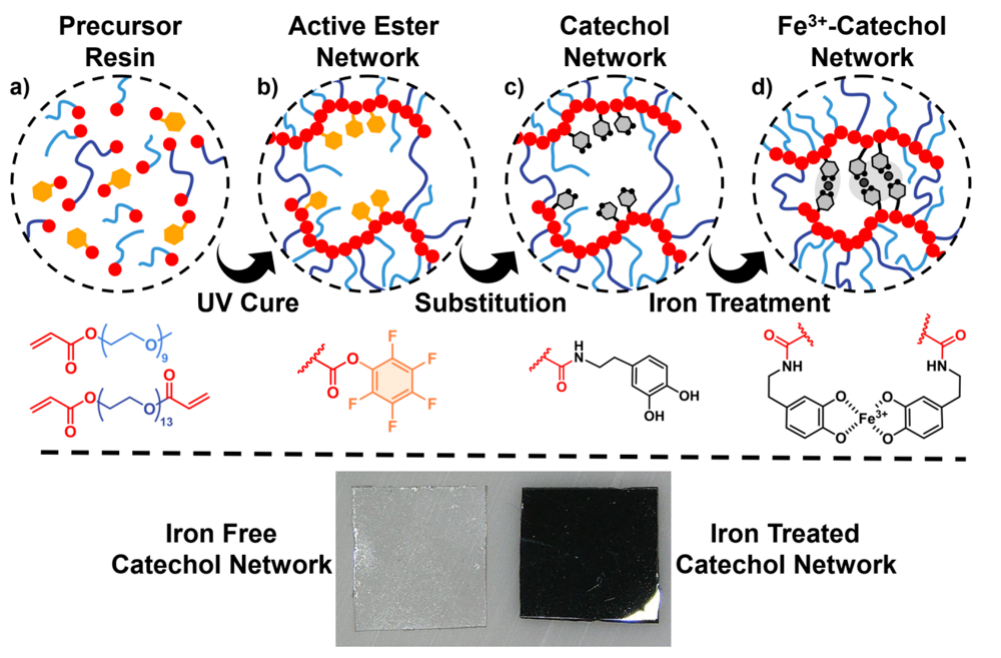
Modular processing of dual cross-linked networks leads to a highly tunable and patternable material following the steps from left to right of the (a) precursor resin, (b) active ester network, (c) catechol substitution, and (d) iron complexation with (bottom) images of catechol networks before and after Fe^3+^ complexation.

For accessing high-stiffness networks, Fe^3+^–catechol bonds are of interest due to their near covalent bond strength and pH-dependent association number (mono vs bis vs tris) with the latter having a significant influence over the resulting cross-link structure.^5,26,31−35^ Recent work from the Valentine group has demonstrated the importance of the Fe^3+^–catechol clustering and ionomeric domains for toughening epoxy-based networks leading to high-stiffness materials in the dry state (*E* ∼250 MPa).^36,37^ In this system, the catechol unit was introduced as a bis(triethylsilyl) protected epoxide during the epoxy/amine network formation reaction followed by deprotection. This combination of a covalent network with a percolating metal–ligand cluster network represents a promising approach to creating stiff, strong, and tough load-bearing polymer networks. Building on this success, there is significant opportunity for extending this design concept to a more modular synthetic strategy based on initial rapid and orthogonal free radical cross-linking followed by secondary functionalization with readily available catechol derivatives such as dopamine, alleviating the requirement for protection/deprotection of catechol moieties^38,39^ (Figure 1). The versatility of radical cross-linking systems and the wide availability of starting materials would also enable the preparation of patterned materials for soft robotics,^40,41^ tissue engineering scaffolds,^42^ and high-strength, functional 3D printed materials.^43^

## Results and Discussion

In this work, we develop a synthetic platform for preparing and patterning high-stiffness networks reinforced by noncovalent Fe^3+^–catechol cross-links in simple, modular network designs.^44^ Previously, we have reported a two-step strategy for the general synthesis of catechol-containing films that uses initial radical cross-linking followed by postpolymerization modification of pentafluorophenyl esters with a variety of commercially available functional amines including dopamine.^44^ Grignon et al.^45^ recently described the use of the same two-step strategy for the modification of RAFT-derived polymers which illustrates the utility of this approach.^44,46^ Application of these techniques to the functionalization of PEG-acrylate cross-linked networks with dopamine-derived catechols and assembly with Fe^3+^ salts leads to the formation of tough noncovalent cross-links that reinforce the network structure (Figure 1). Importantly, the inherent tunability of this strategy allows a wide range of mechanical property changes and increased mechanical stiffness to be obtained. Additionally, through use of soft lithography approaches, we demonstrate the selective patterning of Fe^3+^ incorporation into the network which provides spatial control of metal–ligand cross-link formation and thin film strengthening.

**Scheme 1:**
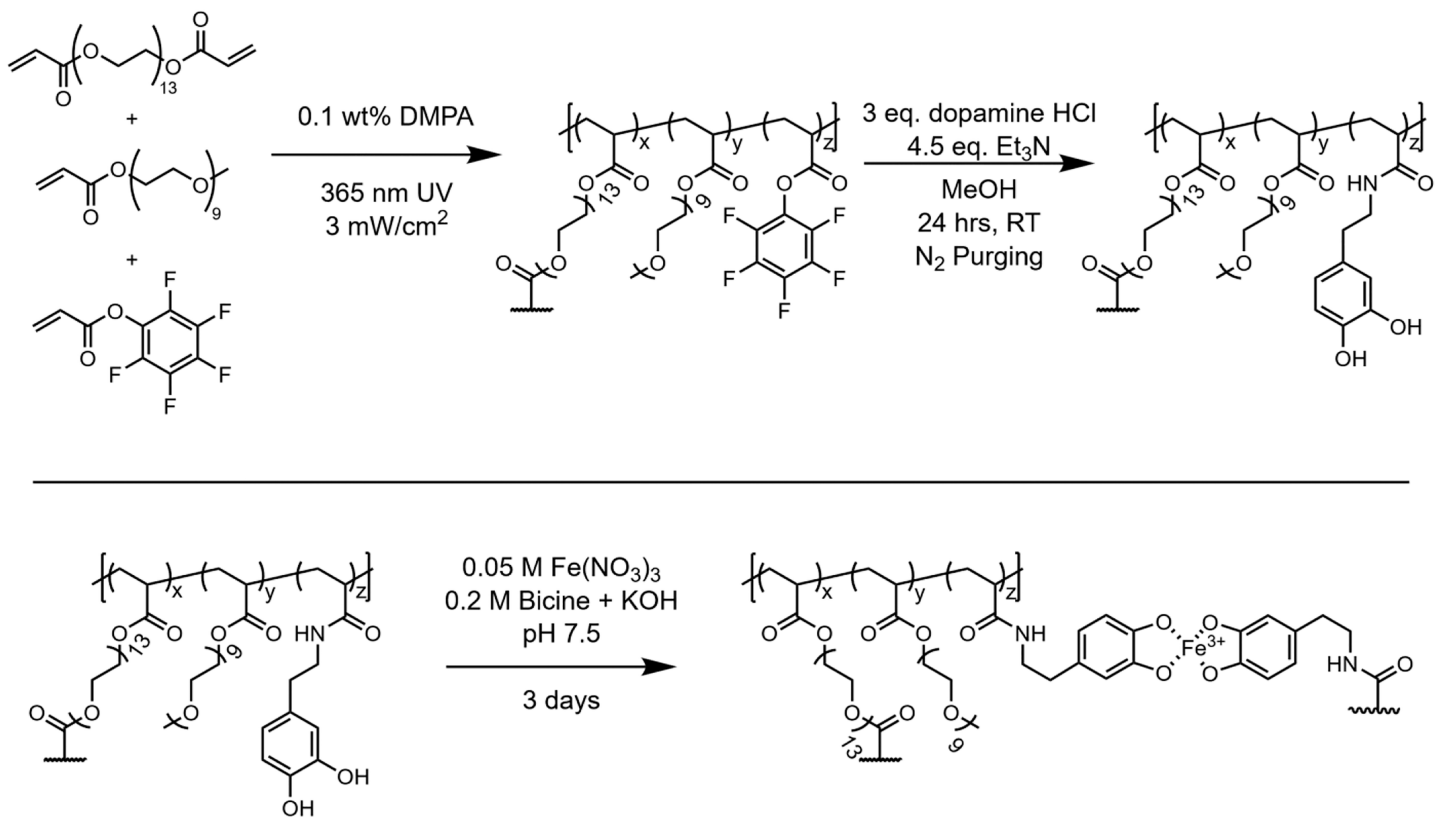
Polymerization of Networks, Substitution of Active Ester Groups with Catechol Moieties, and Subsequent Complexation with Fe^3+^

Polymer networks were prepared according to Scheme 1 from a solvent-free mixture composed of three monomers: an active ester monomer (pentafluorophenyl acrylate, PFPA), a diacrylate cross-linker [poly(ethylene glycol) diacrylate, PEGDA, *M*_n_ 700], and a monofunctional diluent monomer [poly(ethylene glycol) methyl ether acrylate, PEGMEA, *M*_n_ 480]. Initiation under UV irradiation at 365 nm for 2 min using 2,2-dimethoxy-2-phenylacetophenone as the photoinitiator (0.1 wt %, DMPA) led to effective polymerization and cross-linking. Networks were prepared between quartz plates to form robust films, with film thickness controlled by spacers placed between the plates. Key to the success of this strategy was the ability to engineer mechanically tough films that could be swollen in a variety of solvents and at different pH values and dried repeatedly without cracking or damage. To achieve this, the amount of PEGDA cross-linker was maintained at low levels (∼3–4 mol %) in the active ester films to yield soft networks that avoided the observed brittleness of highly cross-linked films (>5 mol %). Through facile radical copolymerization of active ester and PEG-acrylate monomers, a diverse range of materials could therefore be prepared with varied cross-linker content and active ester side chain densities (Tables 1 and 2, and Table S1).

**Table 1:** Library of Iron–Catechol Reinforced Network Compositions (Mixed Cross-Linkers)^a^

network composition			theoretical maximum cross-link density (mmol/cm^3^)			
mol % catechol	mol % PEGDA	mol % PEGMEA	total cross-link density	covalent cross-link density	metal–ligand cross-link density	metal–ligand to covalent cross-link ratio
4.4%	4.4%	91%	0.15	0.1	0.05	0.50
20%	3.9%	76%	0.35	0.1	0.25	2.5
35%	3.5%	61%	0.60	0.1	0.5	5.0
48%	3.2%	49%	0.85	0.1	0.75	7.5
58%	2.9%	39%	1.1	0.1	1.0	10

a Theoretical maximum cross-link density is determined from the combined content of covalent (PEGDA) cross-linking units and metal ligand (Fe^3+^–catechol) linkages divided by two, assuming only bis complexation of catechol moieties.

**Table 2:** Control Samples Based on Covalent-Only Network Cross-Linking^a^

network composition		
mol % PEGDA	mol % PEGMEA	theoretical maximum cross-link density (mmol/cm^3^)
4.5%	95.5%	0.1
12%	88%	0.25
24%	76%	0.50
54%	46%	1.0
100%	0%	1.6

a Theoretical maximum cross-link density is determined from the mmol/cm^3^ content of covalent (PEGDA) cross-linkers in the final network.

**Figure 2:**
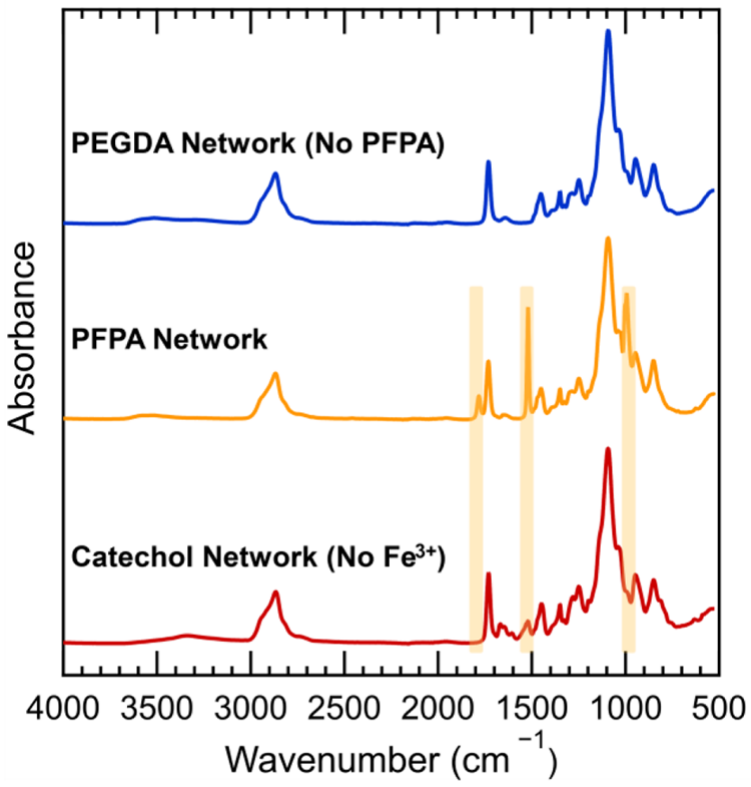
FTIR-attenuated total reflection (ATR) of random copolymer networks. From top to bottom: the blue trace is a 4.5/95.5 mol % PEGDA/PEGMEA network without PFPA monomer incorporation, the yellow trace a 4/61/35 mol % PEGDA/PEGMEA/PFPA network before substitution, and the red trace a PFPA-containing network after reaction with dopamine in MeOH. Key resonances from PFPA (yellow highlighted peaks) at 1780, 1520, and 985 cm^–1^ disappear after substitution with dopamine, with an amide stretching band appearing near 1660 cm^–1^.

As detailed previously,^44^ catechol units were incorporated into these networks via active ester substitution of pentafluorophenyl esters using commercially available dopamine (2-(3,4-dihydroxyphenyl)ethylamine). Films were soaked in a methanol solution of dopamine hydrochloride (excess) for 24 h at room temperature, with triethylamine added to deprotonate the dopamine hydrochloride salt (1.5 equiv of Et_3_N per dopamine HCl). The reaction chamber was degassed with nitrogen, as catechols are known to oxidize in the presence of oxygen and under basic conditions to form quinones which can undergo dismutation and aryloxy cross-linking to form undesirable catechol–catechol covalent cross-links, as well as Michael-type addition with amines to form more complex polydopamine structures.^31,32,35,47^ Monitoring by FTIR (Fourier transform infrared spectroscopy) confirms complete substitution of the active esters with dopamine to form the corresponding amides with no detectable quinone formation. As shown in Figure 2, resonances at 1780, 1520, and 985 cm^–1^ from the pentafluorophenyl ester group disappear after substitution with dopamine, and a concomitant appearance of an amide stretching resonance is observed at ∼1660 cm^–1^.

To illustrate the reproducibility of this process, a wide range of PFPA networks were prepared, and after active ester substitution under an inert atmosphere, all samples were transparent and colorless, indicating little or no catechol oxidation. In contrast, reactions performed in the presence of air consistently yielded brown products, which is indicative of catechol oxidation and cross-linking.^31,32,35,47^ To demonstrate oxidation under controlled conditions, control experiments were performed by intentionally oxidizing films in a solution of NaIO_4_ (0.05 M),^33,48^ which immediately resulted in a change in physical appearance from colorless to orange/red^31,33,48−50^ (see Supporting Information, Figure S1). After catechol substitution, films were dialyzed for 24 h in 0.1 M HCl followed by an additional 24 h dialysis in 1 mM HCl to remove triethylamine salts and residual, unreacted dopamine. The second dialysis step raises the pH of the cross-linked network to be closer to that of the Fe^3+^ complexation solution (pH 7.5), which favors formation of Fe^3+^–catechol bis-complexes.^31,32^

A wide range of conditions were examined for formation of Fe^3+^–catechol complexes within the cross-linked films, and exposure of the catechol-containing networks to an aqueous solution containing 0.05 M Fe(NO_3_)_3_ and 0.2 M bicine buffered with KOH at pH 7.5 was found to be mild and the most reproducible process.^36,37^ This procedure allows soluble iron–bicine complexes to diffuse into the covalently cross-linked films and subsequently exchange with the catechol ligands to form a stable, secondary set of network cross-links based on bis-catecholate iron complexes. Excess iron salts and bicine were removed from films by thoroughly dialyzing the swollen networks in DI water. To maintain mechanical integrity of the films during drying, it was important to employ solvent gradients after the iron treatment and washing steps. This involves slow addition of solvents with decreasing polarity to gradually dehydrate large-surface-area films prior to drying under vacuum. This gradual switch from aqueous to low-polarity organic solvents mitigated cracking from film contraction during drying and rapid solvent switching (see the Supporting Information for details).

**Figure 3:**
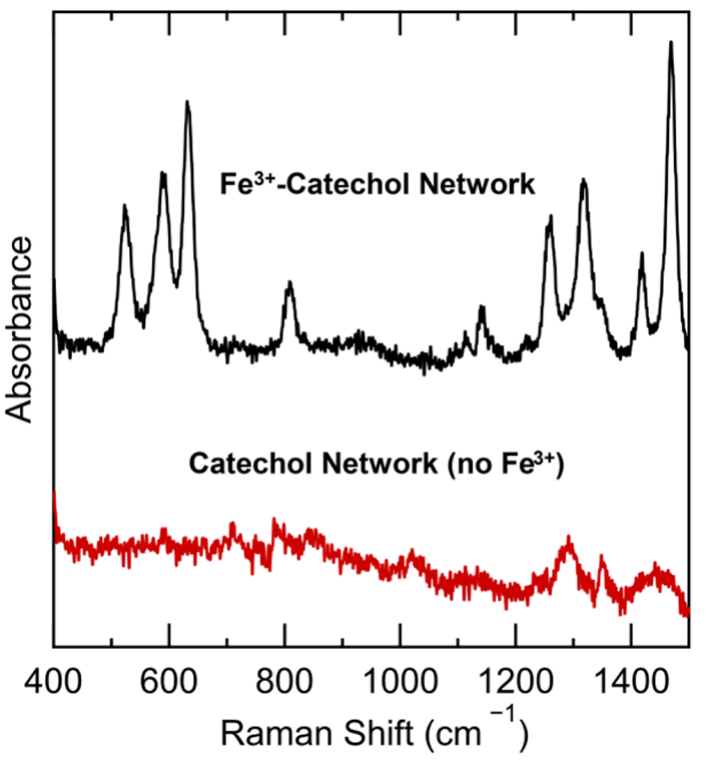
Raman spectra of 3/39/58 mol % PEGDA/PEGMEA/PFPA films after substitution with dopamine. From top to bottom: the black trace is film after complexation with Fe^3+^, and the red trace is the corresponding film without iron treatment. Resonances at 630, 590, and 520 cm^–1^ indicate the presence of Fe^3+^–catechol bis-complexes.

Raman spectroscopy confirmed the primary formation of bis-Fe^3+^–catechol complexes in iron-treated films (Figure 3). Raman signals at 630, 590, and 520 cm^–1^ were observed and are indicative of Fe^3+^–catechol vibrations with signals at 1200–1500 cm^–1^ corresponding to catechol ring vibrations after complexation.^31,37^ Significantly, Raman microscopy imaging of the cross-linked films showed the homogeneous appearance of signals for bis-Fe^3+^–catechol complexes throughout the thickness of the films and were observed to be absent in iron-free catechol films.

Further analysis of the network morphology utilized small-angle X-ray scattering (SAXS) to identify the clustered microstructure of Fe^3+^–catechol units within the bulk films.^36,37^ Prior literature has highlighted the importance of Fe–catechol clustered domains on reinforcing network structure^36,37^ as well as the impact of ionic structures on mechanical properties.^51,52^ Initial SAXS measurements were therefore performed on control PEG networks and precursor films to correlate microstructural changes with variations in cross-linker density, pentafluorophenyl ester incorporation, and Fe^3+^–catechol complexation. Scattering was conducted on both water-swollen and dry films, with swollen structures providing sufficient contrast for measurement (data from dry films is included in the Supporting Information, Figure S11). Film compositions were chosen to be homogeneous in iron content throughout the film cross section, as discussed below, by controlling thickness, Fe^3+^ treatment, and catechol grafting density.

**Figure 4:**
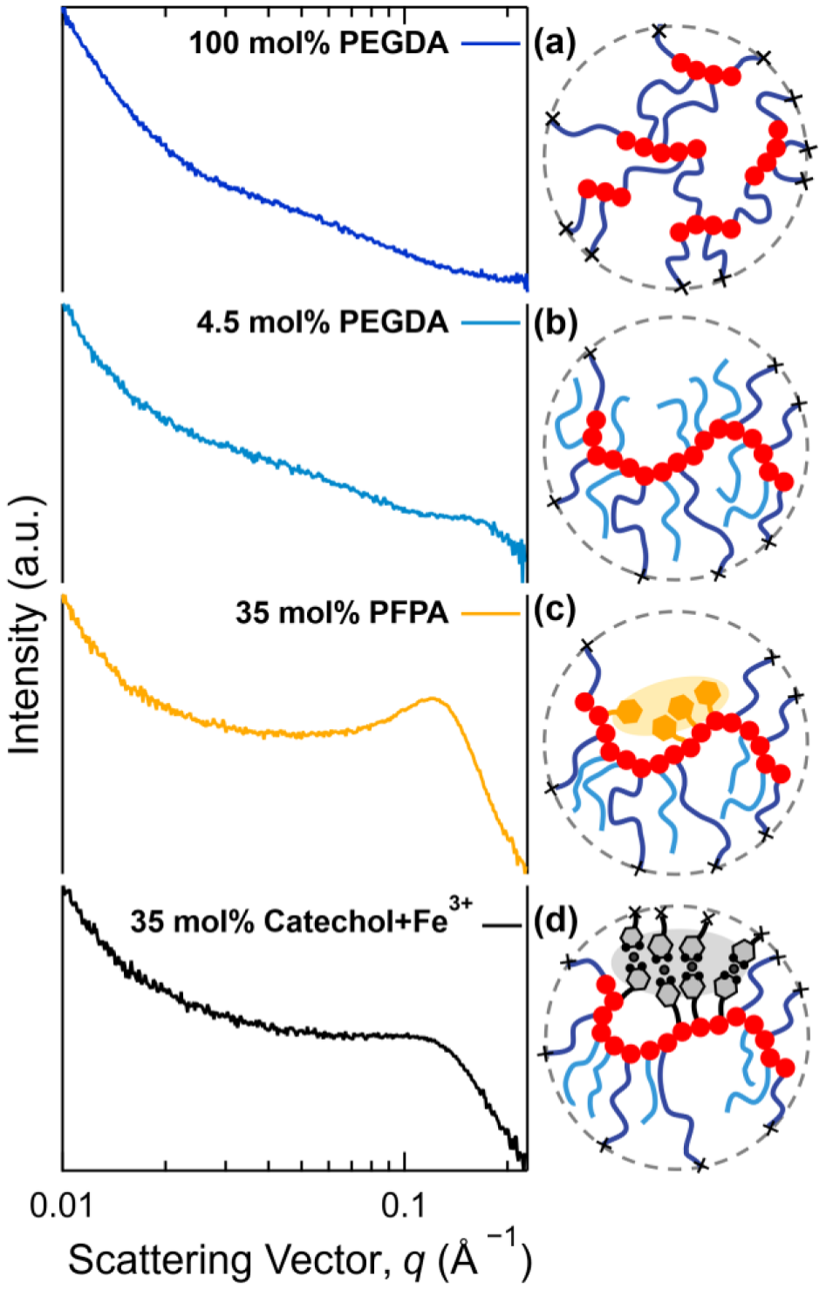
Small-angle X-ray scattering of water-swollen polymer networks and representative cartoons of the polymer microstructure. (a) Scattering of 100 mol % PEGDA network, (b) scattering of 4.5/95.5 mol % PEGDA/PEGMEA network, (c) scattering of 4/61/35 mol % PEGDA/PEGMEA/PFPA network, and (d) scattering of 4/61/35 mol % PEGDA/PEGMEA/catechol network after substitution with dopamine and subsequent Fe^3+^ complexation.

In accord with previous work by Helgeson,^53^ pure PEGDA networks display heterogeneous, starlike microstructures which deviate from ideal network topologies. Key features are observed in the SAXS trace of the swollen, pure PEGDA sample (100 mol % PEGDA, Figure 4a) such as the low *q* upturn from the network and minimal contrast between PEG side chains and short acrylate backbones. Inclusion of PEGMEA as a comonomer results in a brushlike structure with increased acrylate backbone lengths between cross-links and enhanced contrast between the network backbones and side chains (4.5 mol % PEGDA, Figure 4b). This results in the appearance of a shoulder peak at higher *q* in the SAXS trace. Here, scattering from PEG side-chain-rich regions is superimposed on the network scattering of the acrylate backbone with minimal other deviations from the pure PEGDA scattering patterns. Such results suggest qualitatively similar levels of heterogeneity across the control samples and are comparable to expected literature morphologies.^53^

Significantly, introduction of the PFPA comonomer leads to the appearance of a peak in SAXS traces, corresponding to a feature of approximately 5 nm in length scale for water-swollen samples (Figure 4c). This feature is attributed to the correlation length between PFPA-rich domains resulting from the hydrophobic character of the pentafluorophenyl ester coupled with the reactivity ratios for PFPA and PEG acrylates (*r*_PFPA_/*r*_PEG-acrylate_ of 1.4/0.22) leading to nonstatistical incorporation of PFPA repeat units along the backbone.^54^ These PFPA-rich domains serve as a template for catechol installation with Fe^3+^-treated films forming clustered domains of Fe^3+^–catechol complexes at a comparable length scale to initial PFPA templating (i.e., ∼5 nm) as seen in the shoulder of the swollen, iron-treated film (Figure 4d). The formation of these domains is further reinforced by a decrease in feature size in SAXS when the samples are dried (Figure S11). For all samples, a lack of crystalline microstructural features in wide-angle X-ray scattering was observed and indicates an amorphous structure (Figure S12).^36,37^

**Figure 5:**
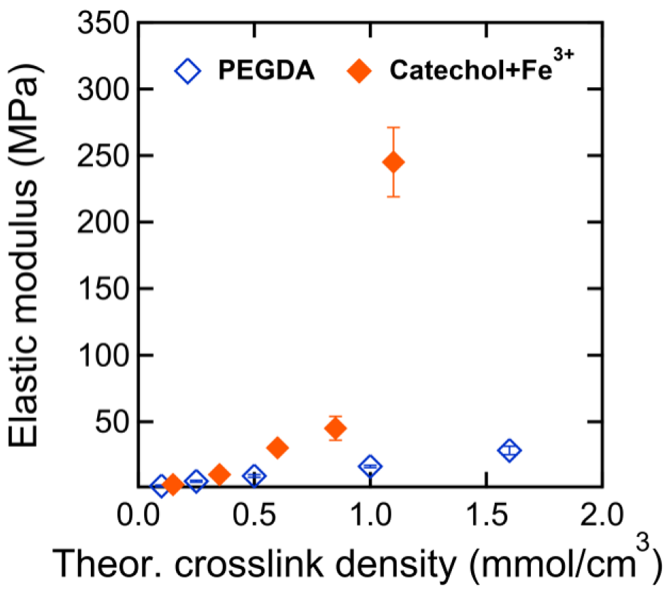
Young’s moduli, *E*, from beam bending measurements of dry samples vs theoretical maximum cross-linking density (mmol/cm^3^) for catechol-free PEGDA networks (unfilled blue diamonds) and Fe^3+^–catechol networks with constant PEGDA covalent cross-linker content of ∼3–4 mol % (filled orange diamonds). Error bars represent standard deviations from replicate measurements. Note that some error bars are within the bounds of the markers (see the Supporting Information for specific values).

The ability to control the level of covalent and noncovalent cross-linking permits characterization of homogeneous, bulk network mechanical properties via beam bending to reveal significant trends across both control networks and Fe^3+^–catechol systems. For purely covalent networks, the Young’s modulus increases from 1.7 to 28.4 MPa as the PEGDA content increases from 4.5 to 100 mol % with the concomitant PEGMEA content decreasing from 95.5% to 0%. This behavior is expected for networks with increasing cross-linking density.^55^ Notably, the introduction of Fe^3+^–catechol units correlated with significant increases in elastic moduli. For networks with constant PEGDA cross-linking (∼3–4 mol %), increasing the Fe^3+^–catechol from 4.4 to 58 mol % resulted in an increase in modulus of ∼2 orders of magnitude from 2.7 to 245 MPa, approximately 10× that observed for the purely covalent system (Figure 5). Prior studies have attributed this substantial increase in stiffness to the clustering of Fe^3+^–catechol complexes, which reinforces the existing covalent network framework via formation of ionic domains.^36,37^

The impact of the Fe^3+^–catechol network was further investigated by rheological stress relaxation experiments that show minimal relaxation, suggesting the Fe^3+^–catechol bonds exhibit quasistatic cross-link behavior during the time scale of the beam-bending testing (see the Supporting Information). Thus, the purely covalent networks can be directly compared with these dual cross-linked networks (covalent + Fe^3+^–catechol) on the basis of their theoretical maximum cross-linking density (cf., Tables 1 and 2) which is calculated based on diacrylate cross-linker content and the assumption that all grafted catechol units form bis-Fe^3+^–catechol cross-links (Figure 5). We find that bis-Fe^3+^–catechol networks are much stiffer than even the most highly cross-linked covalent PEGDA networks, despite similarities between their theoretical total cross-linking densities. To determine if the increased stiffness of networks incorporating Fe^3+^–catechol bonds was in part due to oxidation and cross-linking of catechol units, elastic moduli were measured for iron-free samples with 35 and 58 mol % catechol monomers after subjection to oxidative cross-linking by NaIO_4_.^33,48^ It is notable that oxidized films exhibited only modest increases in stiffness from 2.4 to 3.7 and 28 MPa, respectively, which are an order of magnitude lower than corresponding Fe^3+^–catechol networks and are comparable to purely covalently cross-linked films. These values further highlight the importance of metal–ligand complexes and associated ionic interactions for achieving high stiffnesses in these materials. While this data demonstrates the impact of bis-Fe^3+^–catechol complexes, formation of tris-Fe^3+^–catechol complexes is also possible via iron incorporation at elevated pH which may lead to interesting future work as modulation of cross-link functionality may further change mechanical behavior, but this is beyond the scope of the present study.^31^

**Figure 6:**
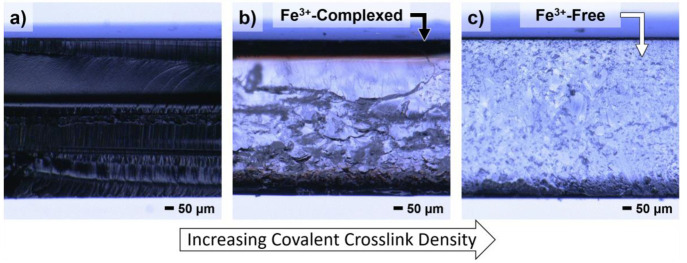
Optical microscopy images of cross sections of films with varied covalent cross-linker content, illustrating differences in iron diffusion rates. (a) 3/39/58 mol % PEGDA/PEGMEA/catechol, (b) 7/33/59 mol % PEGDA/PEGMEA/catechol, and (c) 15/24/61 mol % PEGDA/PEGMEA/catechol.

An important advantage of this two-step approach to dual cross-linked materials is the ability to independently tune the level of catechol incorporation and cross-link density of the initial covalent network. To illustrate the potential of this stepwise strategy, gradients in iron content were programmed into the secondary catechol network by controlling the diffusion rate of the external Fe(NO_3_)_3_ solution into the cross-linked thin films through both covalent cross-linking density and polymer–ion interactions which are both known to slow ion diffusion into water-swollen networks.^56−61^ Significantly, the penetration depth of Fe^3+^ ions into swollen catechol networks was shown to be modulated by changing covalent cross-linking density, catechol grafting density, film thickness, and time. For example, by fixing catechol grafting density and film thickness and varying covalent cross-linking density (i.e., increasing PEGDA content), optical microscopy reveals that iron incorporation into highly cross-linked networks (7+ mol % PEGDA) is substantially slowed relative to more loosely cross-linked networks (∼3–4 mol % PEGDA). As can be seen in Figure 6, tuning of iron diffusion results in Fe^3+^–catechol complex formation that is localized to thin “skin” layers at the external surfaces of the films, with essentially no iron reaching the center of the films. Additional sample specifications are located in Table S1.

**Figure 7:**
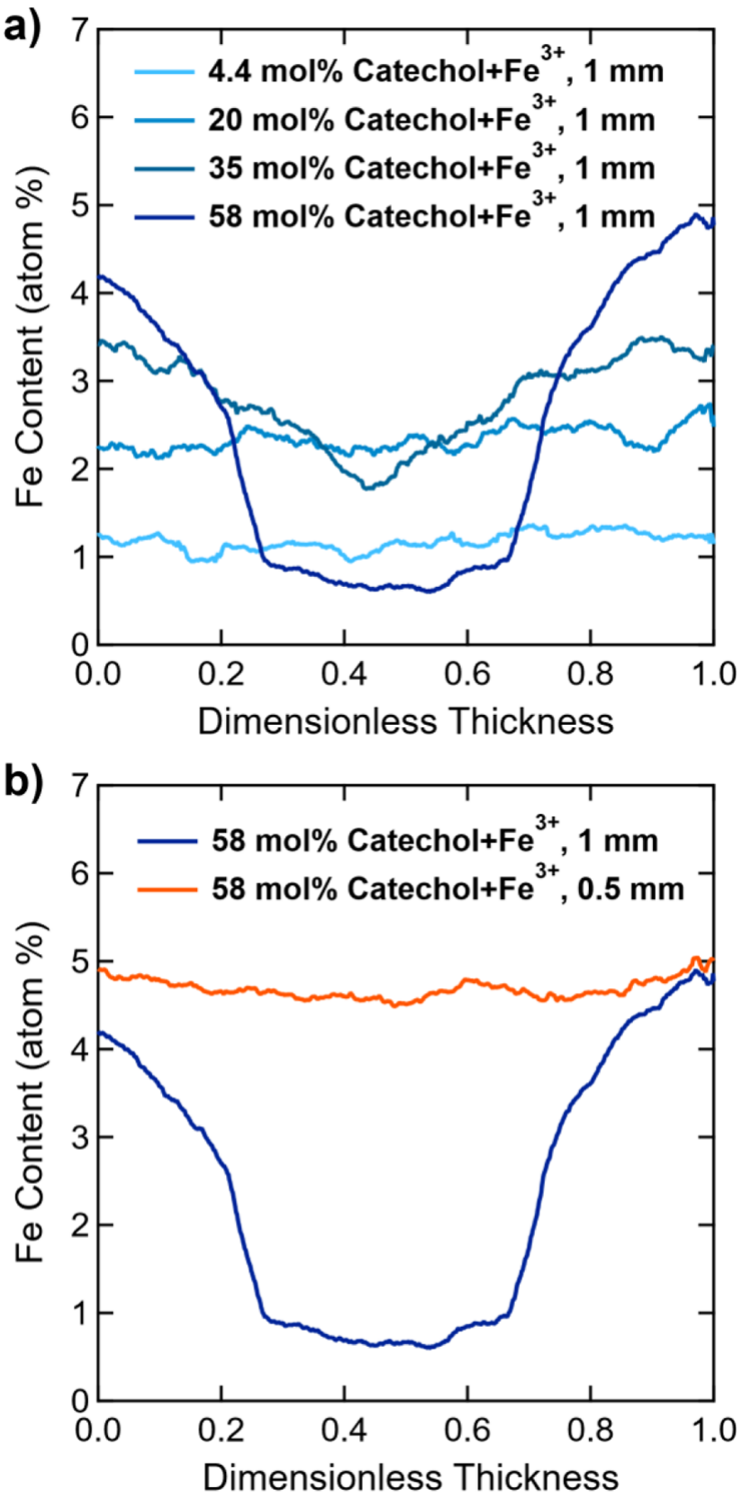
SEM EDS characterization of Fe^3+^ distributions across film thicknesses. (a) Fe^3+^ distribution vs dimensionless thickness for 1 mm thick films with ∼3–4 mol % PEGDA and varied catechol content after exposure to Fe^3+^ solution for 72 h. (b) Comparison of Fe^3+^ distribution vs dimensionless thickness for 0.5 and 1 mm thick films of 3/39/58 mol % PEGDA/PEGMEA/catechol film after exposure to Fe^3+^ solution for 72 h.

Alternatively, at low covalent cross-linking density (e.g., 3–4 mol % PEGDA), increasing the catechol grafting density from ∼4 to 58 mol % results in an increase in the total amount of iron uptake while also hindering ion diffusion into the center of the networks, again forming a gradient in Fe^3+^ concentration. This can be quantified by energy-dispersive X-ray spectroscopy (EDS) line-scans of SEM cross sections which illustrate more homogeneous Fe^3+^–catechol complexation in low-catechol-content samples (≤20 mol % catechol-containing monomers) and more surface-concentrated Fe^3+^-rich domains in networks with increased catechol content (≥35 mol %; constant film thickness of 1 mm and 72 h iron treatment; Figure 7a). Finally, film thickness is also important for controlling homogeneous formation of Fe^3+^–catechol cross-links. For samples with low PEGDA content (∼3 mol %) and high catechol content (58 mol %), homogeneous Fe^3+^ distributions could be achieved by reducing film thickness from 1 to 0.5 mm during the same 72 h iron treatment (Figure 7b). The further impact of iron diffusion time scales and surface-driven gradients can be seen in the Supporting Information (Figures S7 and S8) and clearly illustrates that Fe^3+^–catechol formation can be controlled by structural parameters to give a wide variety of mixed cross-link and gradient systems.

**Figure 8:**
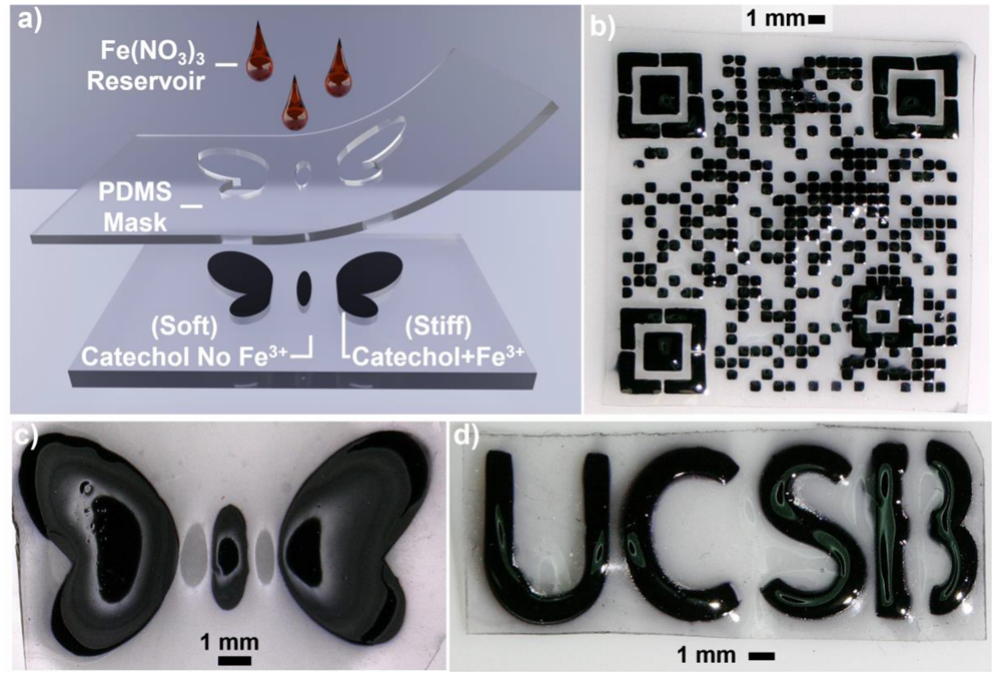
(a) 3D rendering of the generalized schematic of the Fe^3+^ patterning approach. (b–d) Images of catechol-containing films (3/39/58 mol % PEGDA/PEGMEA/catechol) after selective patterning with Fe^3+^ through soft lithography styled masking displaying (b) a QR code, (c) a butterfly, and (d) a demonstration of text.

**Figure 9:**
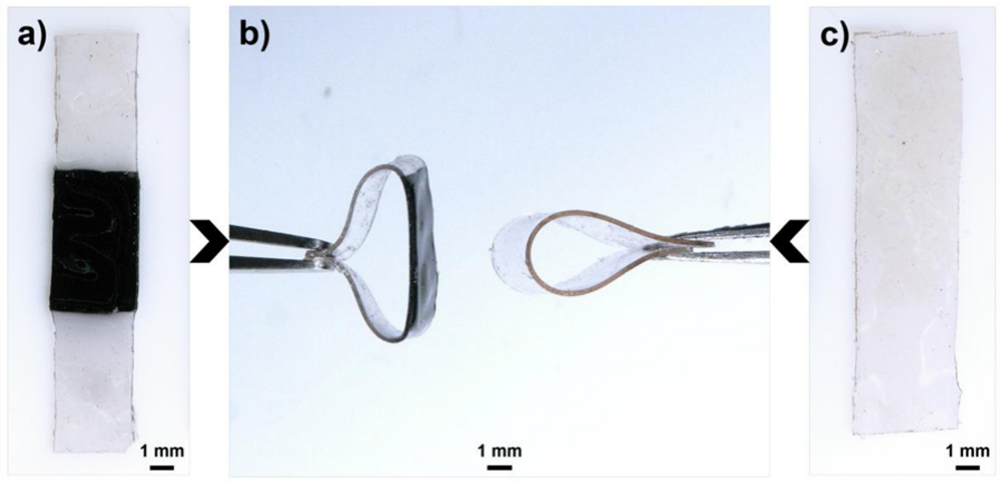
(a) Continuous film of 3/39/58 mol % PEGDA/PEGMEA/catechol with a central section functionalized with Fe^3+^–catechol groups. (b) Comparison of folded film strips between iron-patterned and iron-free networks. (c) Continuous film of 3/39/58 mol % PEGDA/PEGMEA/catechol with no patterning or introduction of Fe^3+^–catechol groups.

This ability to prepare gradient Fe^3+^–catechol network structures also opens up the possibility of controlling the patterning of Fe^3+^–catechol domains in the *X*- and *Y*-directions. Soft lithography solution masking techniques coupled with controlled Fe^3+^ diffusion allow 3D network films to be prepared with a diversity of hard (Fe^3+^–catechol) and soft (catechol only) network patterns.^40,62,63^ To illustrate the utility of this strategy, swollen networks were covered with a PDMS mask that preferentially exposed distinct regions of the network surface to Fe^3+^ via a reservoir of buffered Fe(NO_3_)_3_ solution (Figure 8a). As iron diffusion is relatively slow in networks with high catechol grafting densities, this approach allows for control of feature sizes at resolutions of ∼700 μm (cf., QR code in Figure 8b). Representative patterned films can be seen in Figure 8b–d as well as the Supporting Information Movies S1 and S2. Patterned sections of catechol-containing films further display increased rigidity relative to untreated sections of material. For example, as observed in Movie S1, the wings of the butterfly shown in Figure 8c are significantly stiffer than the Fe^3+^-free matrix. To further demonstrate the dramatic change in mechanical properties, a simple bending demonstration was conducted comparing a patterned Fe^3+^-containing film and a corresponding precursor film (Fe^3+^-free) (Figure 9 and Movie S2). As can be seen in Figure 9, the catechol-containing film is flexible and bends evenly along its length. In direct contrast, the film with a central section containing Fe^3+^-complexed catechol groups bends asymmetrically with the Fe^3+^-containing region being significantly stiffer than the precursor film. This approach for patterning stiff regions in a functional network may further provide a modular tool for generating composite-like soft materials,^64^ as well as a method for patterned adhesion from free catechol groups in iron-free sections.^65^ Additional details about the techniques used for soft lithography solution masking are provided in the Supporting Information.

## Conclusion

In this work, a modular approach for preparing mechanically tunable materials using robust stepwise synthesis of dual cross-linked networks is presented. The versatility of free radical cross-linking of vinyl monomers for preparation of an initial functionalized network is combined with the ease of postmodification using active ester substitution to form catechol-grafted networks without the need for protection/deprotection strategies. These catechol functionalized networks can then be mechanically reinforced with Fe^3+^–catechol cross-links through simple solution processing to yield tough materials with stiffnesses that exceed those of covalently cross-linked PEGDA networks by up to 2 orders of magnitude. Furthermore, soft lithography solution masking techniques can leverage controlled iron diffusion into catechol networks to spatially pattern 3D films with hard–soft regions with sub-millimeter feature sizes and to prepare novel gradient systems. The user-friendly nature of this two-step strategy for preparing mechanically tunable and patterned dual, covalent, and metal–ligand cross-linked networks enables the development of materials with enhanced properties for soft robotic and bioinspired composite applications.

## Experimental Section

Experimental details are described in the Supporting Information.

## Supporting Information Available

Experimental methods, sample property tables, and additional patterning methods (PDF)
Movie S1: Wings of the butterfly shown in Figure 8c are shown to be significantly stiffer than the Fe^3+^-free matrix (MP4)
Movie S2: Bending Comparison of Iron Patterned vs Iron Free Films (MP4)
