# Supplementary material for: Modular Synthesis and Patterning of High-Stiffness Networks by Postpolymerization Functionalization with Iron–Catechol Complexes
Source: Macromolecules. 2023 Mar 15;56(6):2268–76. doi: 10.1021/acs.macromol.2c02561 (PMC10064740; doi:10.1021/acs.macromol.2c02561)
Supplement: Supplementary file 1 — ma2c02561_si_001.pdf [file ma2c02561_si_001.pdf]

Modular Synthesis and Patterning of High Stiffness Networks by Post Polymerization Functionalization with Iron-Catechol Complexes

Declan Shannon[a,e], Joshua D. Moon[a,b], Christopher W. Barney[b,c,e], Nairiti J. Sinha[b,e], Kai-Chieh Yang[b], Seamus D. Jones[b], Ronnie V. Garcia[d], Matthew E. Helgeson[b,e], Rachel A. Segalman[a,b,d,e], Megan T. Valentine[c,e], Craig J. Hawker[a,d,e*]

Author Addresses

[a]Materials Department, University of California Santa Barbara, Santa Barbara, CA 93106-5050

[b]Department of Chemical Engineering, University of California, Santa Barbara, Santa Barbara, CA 93106-5080

[c]Department of Mechanical Engineering, University of California, Santa Barbara, Santa Barbara, CA 93106-5070

[d]Department of Chemistry & Biochemistry, University of California Santa Barbara, Santa Barbara, CA 93106-9510

[e]Materials Research Laboratory, University of California Santa Barbara, Santa Barbara, CA 93106-5121

Author emails:

Declan Shannon – dpshannon@ucsb.edu

Joshua Moon - jdmoon@ucsb.edu

Christopher Barney - barneyc@ucsb.edu

Nairiti Sinha - sinha@ucsb.edu

Ronnie Garcia - ronnie@ucsb.edu

Seamus Jones - seamusdjones@ucsb.edu

Kai-Chieh Yang - kai-chieh@ucsb.ed

Matt Helgeson - helgeson@ucsb.edu

Rachel Segalman - segalman@ucsb.edu

Megan Valentine - valentine@engineering.ucsb.edu

Craig J. Hawker – hawker@mrl.ucsb.edu

Supporting Information

Table of Contents

1. Materials & Instrumentation 2

2. Synthesis of PFPA Monomer 2

3. Polymer Network Synthesis 3

4. Deswelling of Large Area Films 3

5. Bulk Iron Treatment of Catechol Films 3

6. Optical Microscopy Images of Fe^{3+}-Catechol Films with High PEGDA Content 4

7. Oxidation of Control Samples 4

8. Fe^{3+} Patterning of Catechol Films..5

9. Rheological Experimental Procedure 6

10. Beam Bending Methods & Summary Data 8

11. SEM EDS Characterization of Fe^{3+} Content..10

12. SAXS & WAXS Experimental Methods & Supplementary Data 11

1. Materials & Instrumentation

All chemicals were purchased and used as received. Poly(ethylene glycol diacrylate) (PEGDA, M_n 700, with inhibitor), poly(ethylene glycol methyl ether acrylate) (PEGMEA, M_n 480, with inhibitor), pentafluorophenol, acryloyl chloride (with inhibitor), dopamine hydrochloride salt, triethyl amine, and DMPA (2,2-dimethoxy-2-phenylacetophenone), were purchased from Sigma Aldrich and used as received. Bicine was purchased from TCI, and $Fe(NO_3)_3$ (nonahydrate, ACS reagent Grade) was purchased from Fischer Scientific (Acros Organics). Isopropanol, hexanes, methanol, and other solvents were procured from Sigma Aldrich and used as received without further drying or distillation, with the exception of DCM used in PFPA synthesis, which was dried via a PureSolv MD-5 dry solvent system.

Fourier Transform Infrared Spectroscopy with Attenuated Total Reflection (FTIR-ATR) analysis of polymer films was conducted on a Thermo Nicolet iS10 spectrometer equipped with a diamond ATR accessory using 64 scans. FTIR measurements were taken at room temperature under ambient atmosphere. Data were corrected using automatic baseline correction.

Raman Spectroscopy was conducted on cut film interior surfaces using a Horiba Jobin Yvon instrument with confocal microscopy and a 632 nm laser. Hole and slit sizes were both set to 500 microns, with 20 accumulations at 2 seconds each.

Optical microscopy images were taken on samples using a Keyence VHX-5000 optical (digital) microscope. Surface illumination was used unless otherwise specified.

Nuclear Magnetic Resonance (NMR) Spectroscopy measurements were collected on a Bruker 400 MHz NMR-Spectrometer in deuterated chloroform ($CDCl_3$).

PDMS sheeting used in masking procedures was obtained from B & J rubber products with mylar backing and a PDMS sheet thickness of 250 microns. Sheets were cut to the desired geometry using a Trotec Speedy 100 laser cutter. Acrylic panels and holders were laser cut using the same equipment.

2. Synthesis of PFPA Monomer

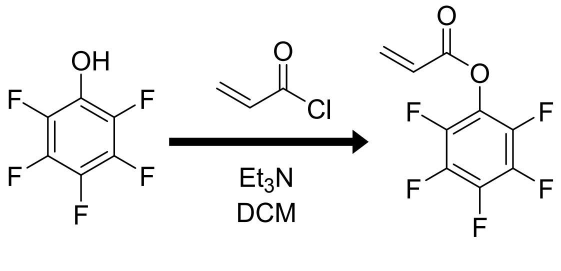

Scheme 1. Synthesis of pentafluorophenyl acrylate (PFPA) monomer.

A revised version of prior synthetic procedure[1] was followed for PFPA synthesis. In short, pentafluorophenol (50 g, 0.27 mol) and Et_3N(40 mL, 0.29 mol) were dissolved in anhydrous DCM (700 mL) and cooled to 0 °C. In a separate flask, acryloyl chloride (24.5 mL, 0.303 mol) was dissolved in 50mL of anhydrous DCM and was added dropwise to cooled pentafluorophenol solution. The mixture was stirred and allowed to warm to room temperature overnight. The reaction was washed with 1M HCl (3x 300mL), then washed with concentrated $NaHCO_3$ (2x 300mL), and dried over $MgSO_4$. The organic phase was then filtered through a layered plug of basic alumina and charcoal and allowed to sit with additional charcoal for 3 hours to remove yellow coloration, filtered, and then concentrated to obtain a clear oil (75% yield). 6.70 (d, *J*= 17.5Hz, 1 H), 6.36 (dd, 1 H), 6.18 (d, *J*=10.5 Hz, 1 H)

3. Polymer Network Synthesis

In a representative procedure of network preparation, DMPA as photoinitiator, PEGMEA, PFPA, and PEGDA were mixed and sonicated for 2 minutes. Monomer resin was dispensed between two quartz plates, with film thickness controlled by steel spacers placed between the plates. Samples were irradiated on one side with 365 nm UV (3 mW/cm^2 power density) for 2 minutes to crosslink the networks. Samples containing PFPA were then immediately placed into a degassed methanol solution containing excess dopamine hydrochloride salt (3 equivalents relative to PFPA), and Et_3N (1.5 equivalents relative to dopamine) was added to begin the catechol substitution. The reaction was gently stirred at room temperature under nitrogen sparging for 24 hours, and films were then placed in increasingly dilute HCl washes for 24 hours (0.1 M and 1 mM) to neutralize unreacted starting material and gradually adjust pH. Swollen films were stored in dilute HCl (1 mM).

4. Deswelling of Large Area Films

To prepare large area films for patterning and mechanical testing, a solvent deswelling procedure was developed. Films swollen in aqueous solutions frequently cracked during direct dehydration under vacuum, so a gentle solvent gradient was developed to slowly remove water from the networks. Water swollen films were placed into a solution with a 3:1 volumetric ratio of DI water and isopropanol (IPA). IPA was gradually added to achieve a 2:1, 1:1, 1:2, and finally 1:3 volumetric ratio of water to IPA with 30 minutes to 1 hour between solvent additions depending on film thickness. Films were then transferred to pure IPA, and hexanes was added stepwise to achieve the same volumetric ratios. Films were then removed from the mixed organic solvent solution and dried directly in vacuum.

5. Bulk Iron Treatment of Catechol Films

Catechol substituted films were removed from their 1mM HCl storage solutions and blotted dry to remove excess solution. Films were then immediately transferred to a solution of 0.05 M $Fe(NO_3)_3$ and 0.2 M Bicine buffered with KOH to pH 7.5. Solution volume was adjusted to be in significant excess (>5×) of the iron content need to fully crosslink films. Unless otherwise noted, bulk samples were left in the iron treatment solution for 3 days. Samples were then removed, blotted dry, and placed into deionized (DI) water to remove residual salts. Films were dialyzed for 3 days with daily exchange of the DI solution, followed by the deswelling procedure described above.

6. Optical Microscopy Images of Fe^{3+}-Catechol Films with High PEGDA Content

Optical microscopy images in the main text (Fig. 6) were taken on cross-sectioned samples using a Keyence VHX-5000 optical (digital) microscope. Mixed transmission and surface illumination was used to highlight dark, opaque regions that were complexed with iron and more transparent regions that were relatively iron free. Samples were noted to have decreasing iron intrusion depths with increasing covalent crosslinker density at high catechol grafting densities.

Table S1: Network compositions for high covalent crosslinker content films. Note, metal-ligand crosslinking ratio targets were not achieved through the bulk of the film due to limited iron diffusion.

**Network Composition**			**Theoretical Maximum Crosslink Density (mmol/cm^3)**			**Metal-Ligand to Covalent Crosslink Ratio**	**Iron Treatment Duration**
mol% Catechol	mol% PEGDA	mol% PEGMEA	Total Crosslink Density	Covalent Crosslink Density	Metal-Ligand		

					Crosslink Density		
58%	3%	39%	1.10	0.10	1.00	10	6 days
59%	7%	33%	1.25	0.25	1.00	4.0	3 days
61%	15%	24%	1.50	0.50	1.00	2.0	3 days

7. Oxidation of Control Samples

Methods for Catechol Film Oxidation

To conduct control studies of intentionally oxidized films, a 0.05 M solution of $NaIO_4$ was prepared. Catechol containing films were removed from their 1 mM HCl dialysis, blotted dry, and placed in significant excess of the periodate solution (>5× excess mol. catechol groups). Samples were allowed to oxidize for 4 days, removed, and dialyzed in DI water for 24 hours before deswelling and drying. Note, caution should be exercised when handling strong oxidizers. Upon exposure to oxidizing conditions, films were noted to have an immediate color change from transparent to a light orange or yellow hue, deepening to the orange/red hues observed in Figure S1.

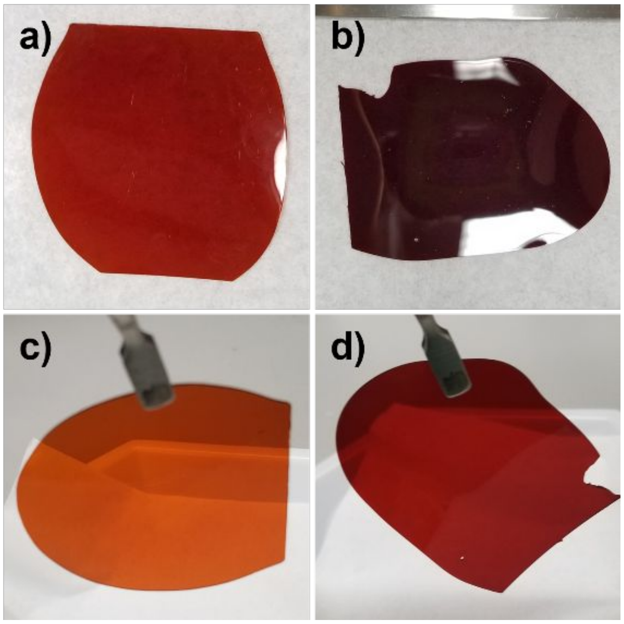

Figure S1: Images of catechol films after oxidation with $NaIO_4$. (a) 3.5 mol% / 35 mol% / 61 mol% PEGDA/catechol/PEGMEA network after oxidation with $NaIO_4$, (b) 2.9 mol% / 58 mol% / 39 mol% PEGDA/catechol/PEGMEA network after oxidation with $NaIO_4$ (c-d). Oxidized films held vertically to demonstrate transparency.

8. Fe^{3+} Patterning of Catechol Films

Patterning of iron-catechol domains could be conducted through a modified literature procedure from a solution-soaked filter paper[2] or directly from solution. For patterning from a soaked filter paper, catechol containing films were removed from a 1 mM HCl storage solution, placed against a glass slide and blotted dry of excess solution. The PDMS mask was then adhered to the film, completely covering the sample, and was then secured to a glass substrate to form an effective seal and prevent iron solution intrusion. The edges of the mask were then secured to the slide using Kapton tape to further prevent leakage. Next, a stack of 4 sheets of filter paper, soaked in the $Fe(NO_3)_3$ solution, was gently pressed against the masked catechol film. The entire assembly was

then secured with a top glass slide and fixed in place with binder clips. The assembled patterning holder was then submerged in the iron solution for the desired patterning duration.

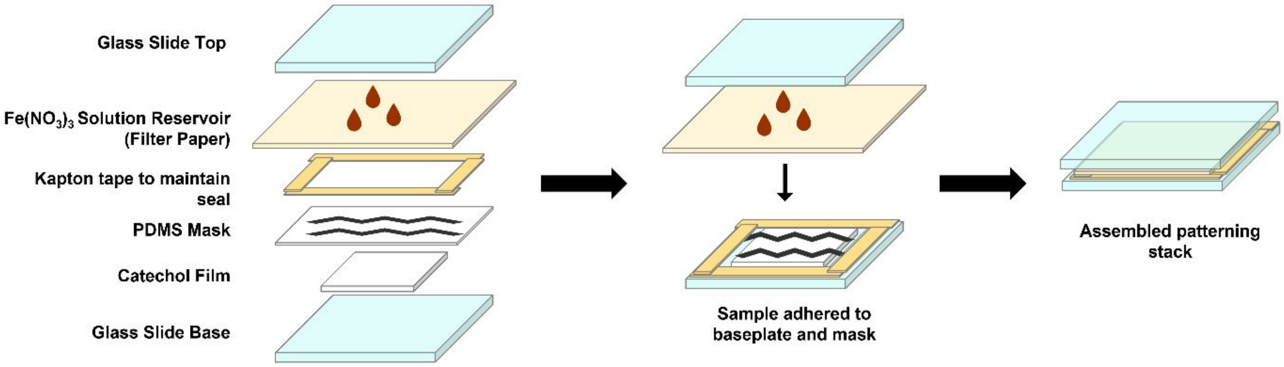

Figure S2: Graphic depicting the components and setup for patterning catechol films from a soaked filter paper.

To pattern samples directly from solution, a similar PDMS masking approach was used, and films could be patterned from both sides. To pattern films from solution, a porous, rigid sample holder (Figure S3A) was prepared from laser cut acrylic sheeting to allow the solution to reach the film surface. Two PDMS masks were laser cut for the front and back of the film, and a PDMS "spacer" of comparable thickness to the catechol film (c.a. 250 microns) was cut to act as a gasket to seal the film between the PDMS masks. To prepare the sample for patterning, one PDMS mask was adhered to the porous acrylic backing, then the PDMS spacer was placed on top of the mask forming a border around the edge of the sample holder (Figure S3B). The catechol film was blotted dry and adhered to the mask on the sample holder, and the top mask was aligned and placed on the surface of the catechol film, sealing with the PDMS spacer below (Figure S3C). The top porous acrylic holder was placed on the assembly (Figure S3D), secured with binder clips, and submerged

in iron solution for the desired time period. The sample could then be removed (Figure S3E), dialyzed, and opened (Figure S3F) to reveal the patterned sample (Figure S3G).

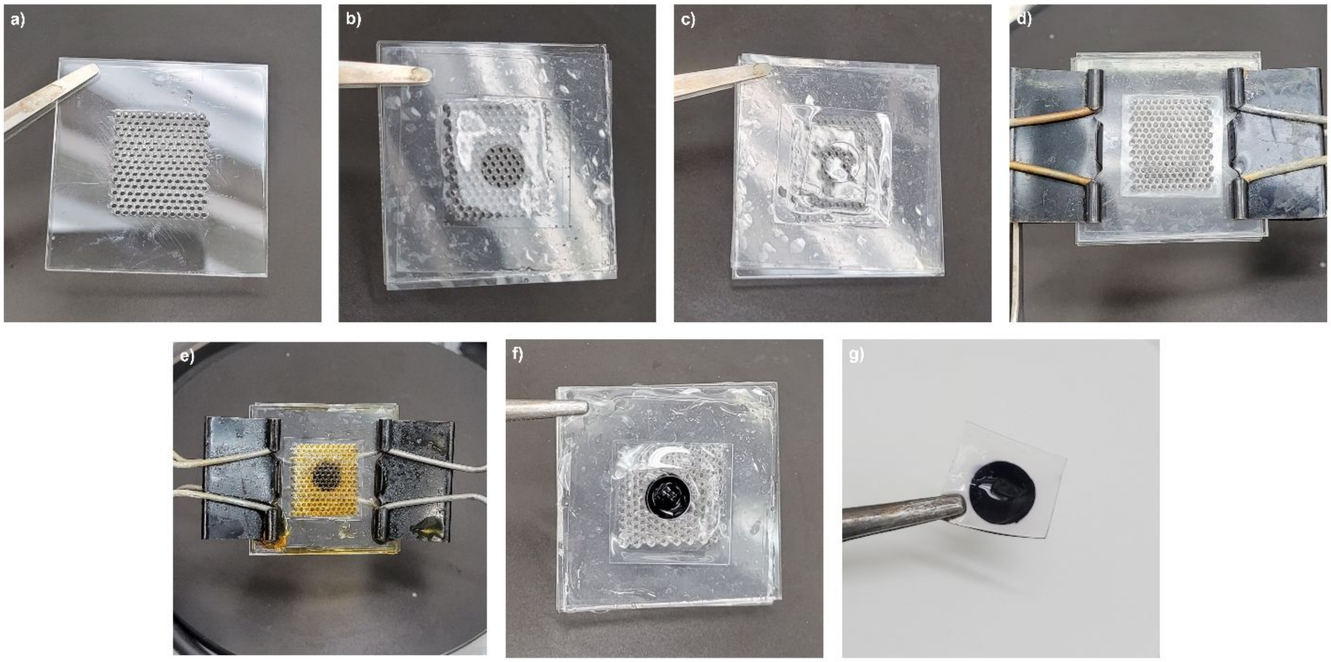

Figure S3: Solution patterning holder assembly. (a) Laser cut, porous acrylic back panel, (b) adhered rear mask and gasket PDMS layers, (c) adhered catechol film and top mask layer, (d) top acrylic panel secured to stack with clips, (e) sample after immersion in iron solution, (f) sample after immersion in iron solution with top acrylic panel removed, (g) solution patterned sample.

9. Rheological Experimental Procedure

Methods

Rheological measurements were made on an ARES-G2 strain-controlled rheometer manufactured by TA instruments. Stainless steel 8 mm parallel plates were used for all measurements. Rheology samples were prepared by using an 8 mm diameter die-punch to prepare flat, circular samples from

vacuum dried films. Samples were placed on the rheometer and compressed at 90 °C and 5N of force. During operation all samples were enclosed in a nitrogen-purged oven chamber. Final sample thicknesses were maintained within a range of 0.5 mm. All measurements were made in the 'linear regime' as determined via strain sweep measurements. Temperature-dependent frequency measurements for time temperature superposition were performed as a sequence of experiments with 5 minutes of soak time between steps. To investigate long-time responses of the materials, linear stress relaxation experiments were also performed. The glassy timescale of the material was determined using fitting to the phenomenological Kohlrausch-Williams-Watts (KWW) expression for the time-dependent modulus G(t). Fitting was performed in frequency space as described in our previous work[3] and utilized the 'libkww' library which provides an approximation for the Fourier transform of stretched exponential functions[4].

Results

Rheological testing of Fe^{3+}-catechol networks at elevated temperatures revealed minimal stress relaxation during the timescale of beam bending (nominally ~5s, Figure S4). These results indicate that during the timescale of beam bending testing, Fe^{3+}-catechol crosslinks behave as quasistatic crosslinks rather than dynamic linkages. To further evaluate the amount of stress relaxation relative to the crosslinked plateau modulus of the iron free network, frequency sweeps at varied temperatures were conducted to generate master curves from the time temperature super positions and the rubbery plateau was identified from (Fig. S5). Even at extended timescales (1000s), samples do not relax to the value of the long timescale crosslinking plateau (Figure S5).

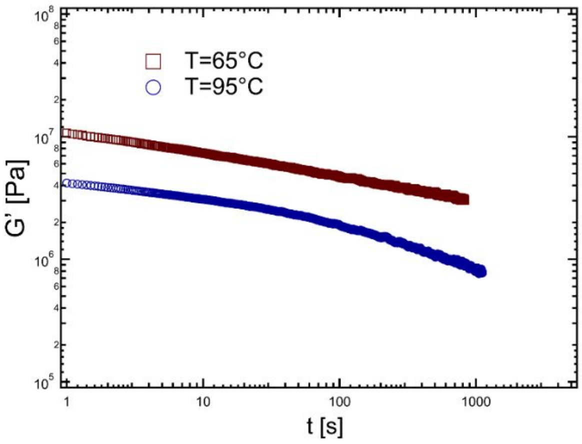

Figure S4: Stress relaxation measurements at elevated temperature of a 2.9/39/58 mol% PEGDA/PEGMEA/catechol network after complexation with Fe^{3+}. Storage moduli of elevated temperature stress relaxation measurements at 65°C (red squares), and 95°C (blue circles) demonstrate minimal stress relaxation during the timescale of beam bending tests (nominally 5s).

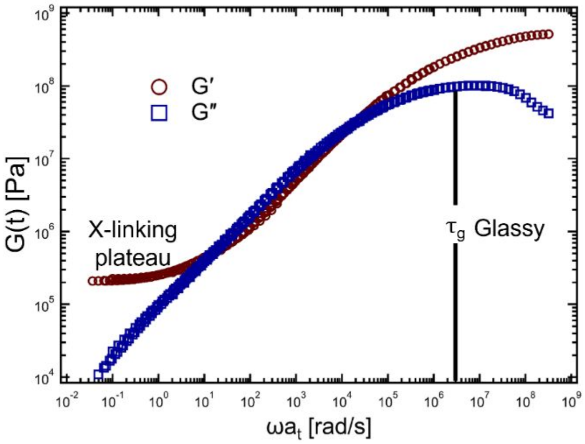

Figure S5: Master curves of storage (red circles) and loss (blue squares) moduli from time temperature superposition *vs.* (shifted) frequency of iron free catechol network, with composition of 2.9/39/58 mol% PEGDA/PEGMEA/catechol, demonstrating expected crosslinking plateau and viscoelastic behavior.

10. Beam Bending Methods & Summary Data

Beam Bending Experimental Procedure

Beam bending was conducted on sectioned samples. Three-point beam bending was performed on a TA.XT Plus Connect Texture Analyzer with a 50 N load cell. Samples were cut into rectangular strips and deformed at a displacement rate of 0.1 mm/s to a turnaround force between 10 and 30 mN depending on sample stiffness. Beam spans were also altered between 15 and 25 mm to control sample stiffness. Summary data and an example plot showing these tests are shown in Figure S6 and Tables S2, S3, and S4. Moduli were calculated according to equation 1, using the sample stiffness k, beam length L, beam width b, and beam height h according to the bending equation for a center loaded beam with simply supported ends[5].

$$E = \frac{kL^3}{4bh^3}$$

Equation 1: Moduli calculation from the bending equation for a center loaded beam.

Beam Bending Diagrams & Supplementary Data

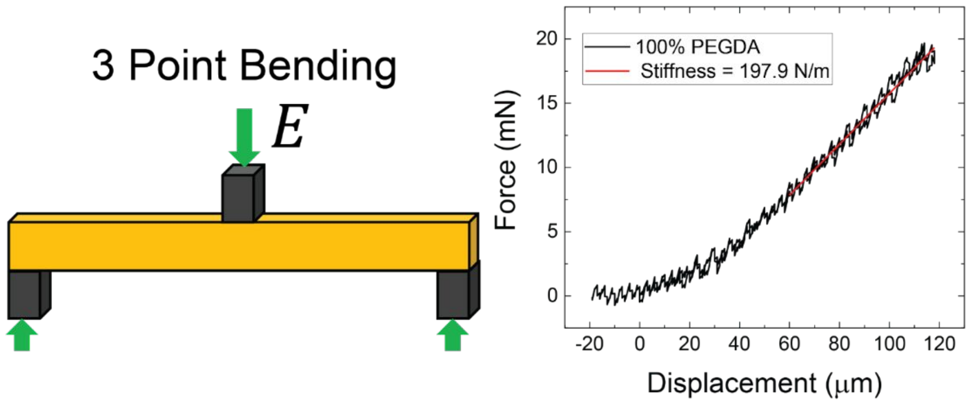

Figure S6: (Left) Diagram of 3-point bending test and (right) example plot of force vs. displacement data (black) and curve fitting of sample stiffness in linear regime (red overlay trace).

Table S2: Beam bending summary data of Fe^{3+}-Catechol films

**Network Composition**			**Theoretical Maximum Crosslink Density (mmol/cm^3)**			**Metal-Ligand to Covalent Crosslink Ratio**	**Young's Modulus, E, (MPa)**	**Standard Deviation (MPa)**	**Number of replicates, n**
mol% Catechol	mol% PEGDA	mol% PEGMEA	Total Crosslink Density	Covalent Crosslink Density	Metal-Ligand Crosslink Density				
4.4%	4.4%	91%	0.15	0.1	0.05	0.5	2.7	--	1*
20%	3.9%	76%	0.35	0.1	0.25	2.5	10.2	1.2	3
35%	3.5%	61%	0.60	0.1	0.5	5.0	30.3	2.0	5
48%	3.2%	49%	0.85	0.1	0.75	7.5	46.5	8.5	6
58%	2.9%	39%	1.1	0.1	1.0	10	245	26	6

*Note: Samples at this composition frequently cracked due to brittle nature of the film, resulting in insufficient surface area for multiple measurements

Table S3: Beam bending summary data for control PEGDA/PEMGEA films

**Network Composition** mol% PEGDA	mol% PEGMEA	**Theoretical Maximum Crosslink Density ($mmol/cm^3$)**	**Young's Modulus, E, (MPa)**	**Standard Deviation (MPa)**	**Number of replicates, n**
4.5%	95.5%	0.1	1.7	0.7	6
12%	88%	0.25	5.2	0.7	4
24%	76%	0.50	9.2	1.0	7
54%	46%	1.0	16.3	0.9	4
100%	0%	1.6	28.4	3.2	7

Table S4: Beam bending summary data of oxidized catechol films

Network Composition			**Young's Modulus, E, (MPa)**	**Standard Deviation (MPa)**	**Number of replicates, n**
mol% Catechol	mol% PEGDA	mol% PEGMEA			
35%	3.5%	61%	3.7	0.4	3
58%	2.9%	39%	28.4	5.5	3

11. SEM EDS Characterization of Fe^{3+} Content

SEM Experimental Procedures

Fe^{3+} distribution through the film was further characterized by scanning electron microscopy (SEM) using a ThermoFisher Apreo C with accelerating voltages of 5 keV. Flat cross sections were prepared by cutting the samples with a blade after quenching in liquid nitrogen. The samples were sputter-coated with 2-3 nm of platinum before imaging. The chemical compositions across the film were analyzed by line scan of energy-dispersive X-ray spectroscopy (EDS).

SEM EDS Analysis of Iron Diffusion Timescale in Bulk and Patterned Films

Iron diffusion gradients could be designed bulk samples (Figure S7) or in surface patterned films (Figure S8) through both temporal and directional control. During iron treatment of bulk catechol films, significant gradients in iron content from the film exterior to interior could be designed through short exposure to the Fe^{3+} solution, with increasing iron content until a homogenous film was achieved. Similarly, in a filter paper patterned sample, iron diffusion gradients could be designed to be directional from a single side of the film and intrusion into the film could be controlled through the duration of patterning.

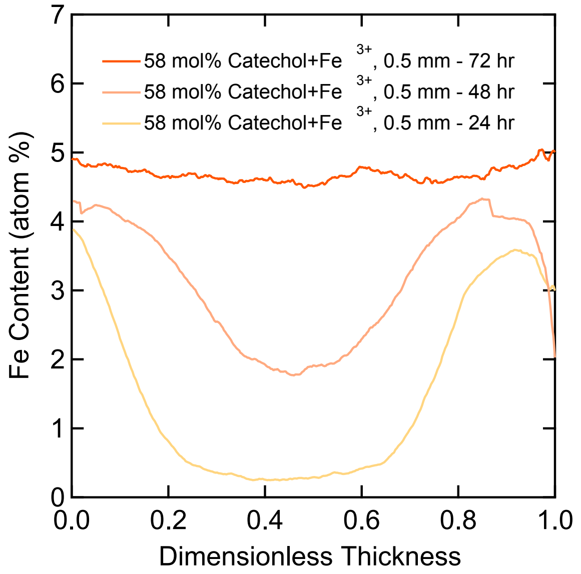

Figure S7: SEM EDS characterization of Fe^{3+} distributions across film thicknesses in a 3/39/58 mol% PEGDA/PEGMEA/catechol film at various timepoints during iron treatment.

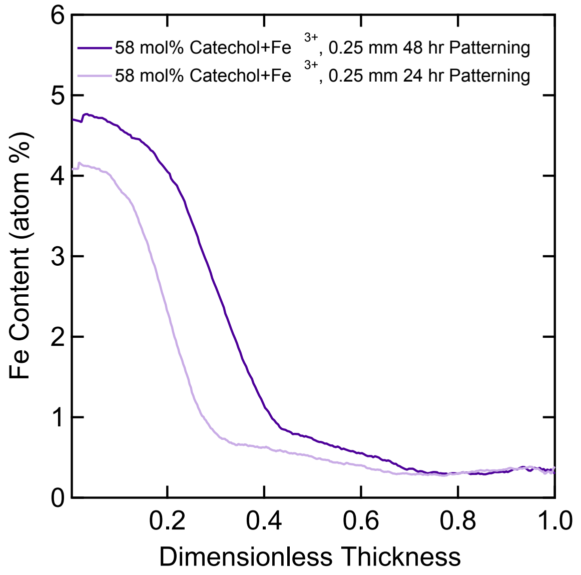

Figure S8: SEM EDS characterization of Fe^{3+} distributions across film thicknesses in a 3/39/58 mol% PEGDA/PEGMEA/catechol film at various timepoints during selective patterning and iron treatment from a saturated filter paper.

12. SAXS & WAXS Experimental Methods & Supplementary Data

Small Angle X-ray Scattering Experimental Procedures

A standard procedure for scattering was as follows. Silver behenate was loaded into a capillary tube and used as a calibration standard. X-ray scattering was conducted using a 1.54 angstrom X-ray beam from a XENOCS Genix 50 W x-ray micro-source, focus size 50 microns, with a XENOCS FOX2D monochromator and Dectris EIGER R 1M detector. X-ray scattering was conducted on each sample for 30 minutes of exposure. Fitting and circular averaging was conducted in Igor pro using the Nika macro program[6]. During SAXS measurements, sections of film in water swollen and dry states were individually placed between protective layers of Kapton tape and affixed to the sample holder. For WAXS measurements, the same instrumentation was used, but no Kapton film was used to cover the material, and dry film sections were suspended in air to reduce noise from Kapton tape layers. All measurements, both SAXS and WAXS, were conducted in triplicate on separate batch of polymer network samples, with no significant deviations in trace morphology observed. Representative data is shown in the main text and in Figures S10, S11, S12, and S13.

SAXS & WAXS Supplementary Data

SAXS measurements of water swollen PFPA or Fe^{3+}-catechol films at higher PFPA/catechol content reveal similar morphological features to films discussed in the main text. Notably for water swollen PFPA samples a significant peak is observed (Figure S9C), corresponding the correlation lengths between PFPA rich regions. Moreover, similar to lower active ester and catechol content

networks, upon substitution and Fe^{3+} complexation in catechol films, a shoulder is observed at comparable length scale to the PFPA rich domains, indicating the formation of clusters (Figure S19D). Scattering of control, catechol networks without iron was alco conducted and is shown in Figure S10.

SAXS and WAXS was also conducted on dried films and demonstrated a decrease in feature size for Fe^{3+}-catechol clusters (cf., shift of peak/shoulder features to the right in Figure S11D & S11F) and a lack of crystalline domains (Figure S12) in iron complexed films. Minimal information was gleaned from dried control PEG-acrylate films and PFPA containing films due to their collapsed structure. WAXS traces of Fe^{3+}-catechol films and control PEG-acrylate films reveal smooth, non-crystalline features indicating the amorphous structure of the catechol clusters.

SAXS Traces of 58 mol% PFPA/Catechol, Water Swollen Films

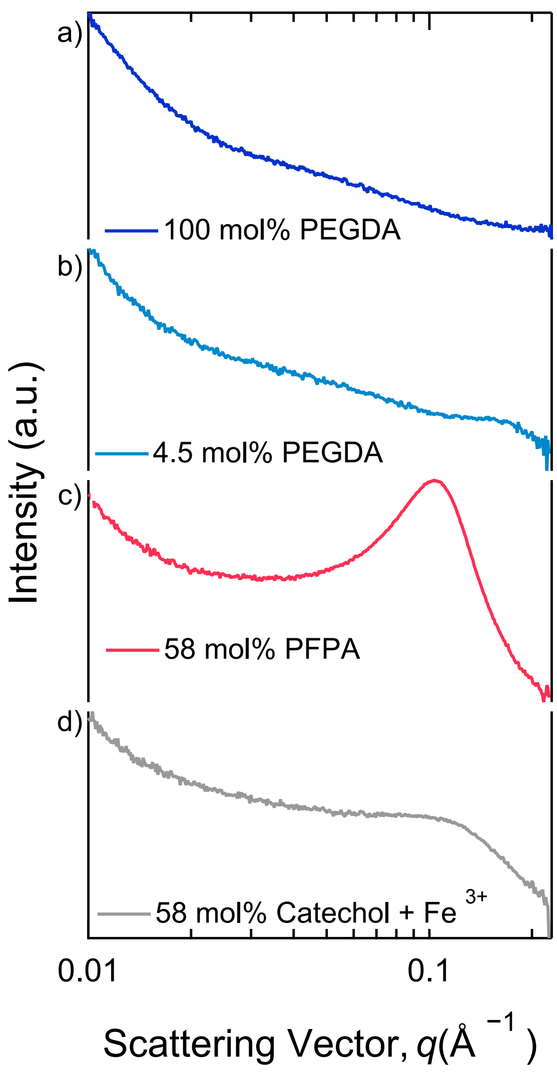

Figure S9: Small angle x-ray scattering of water swollen polymer networks with comparisons between control and 58 mol% PFPA or catechol networks (a) Scattering of 100 mol% PEGDA network, (b) scattering of 4.5/95.5 mol% PEGDA/PEGMEA network, (c) scattering of 2.9/39/58 mol% PEGDA/PEGMEA/PFPA network, (d) Scattering of 2.9/39/58 mol% PEGDA/PEGMEA/catechol network after substitution with dopamine and subsequent Fe^{3+} complexation.

SAXS traces of 1mM HCl solution swollen catechol films without Fe^{3+}

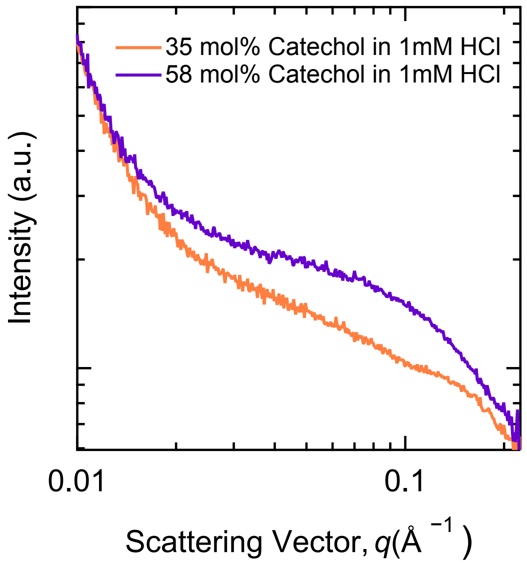

Figure S10: Small angle x-ray scattering of swollen catechol grafted networks in 1mM HCl solution with 3.5/61/35 mol% PEGDA/PEMGEA/catechol (orange) and 2.9/39/58 mol% PEGDA/PEGMEA/catechol (purple)

Dry SAXS Traces of 35 mol% and 58 mol% PFPA/catechol Films

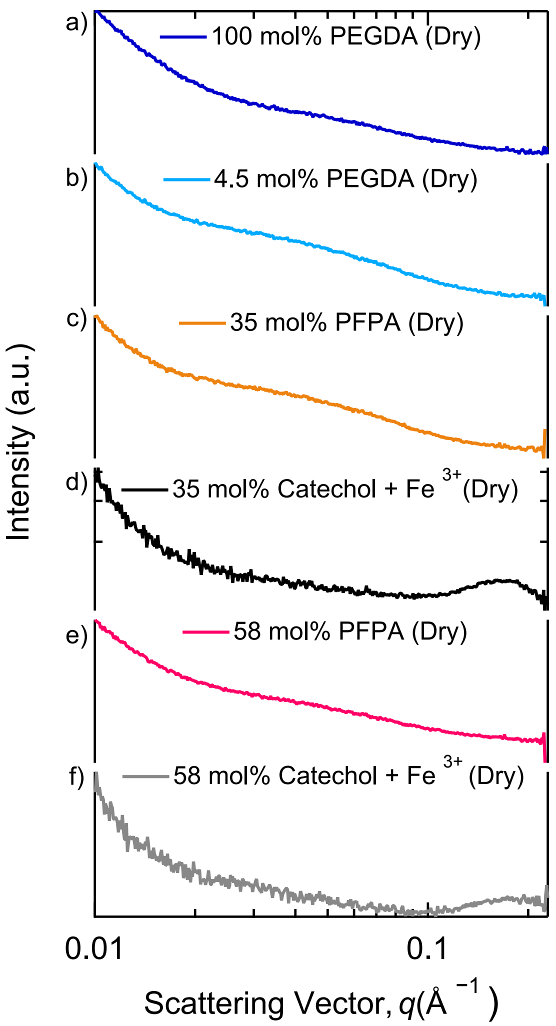

Figure S11: Small angle x-ray scattering of dry polymer networks with comparisons between control, PFPA, and Fe^{3+}-catechol networks. (a) Scattering of 100 mol% PEGDA network, and (b) scattering of 4.5/95.5 mol% PEGDA/PEGMEA network. (c) Scattering of 4/61/35 mol% PEGDA/PEGMEA/PFPA network before dopamine substitution and (d) scattering after substitution and Fe^{3+} incorporation to form a 4/61/35% PEGDA/PEGMEA/catechol-Fe^{3+} film. (e) Scattering of a 2.9/39/58 mol% PEGDA/PEGMEA/PFPA network and (f) after substitution with dopamine and subsequent Fe^{3+} complexation.

WAXS Traces of 35 mol% and 58 mol% catechol Films

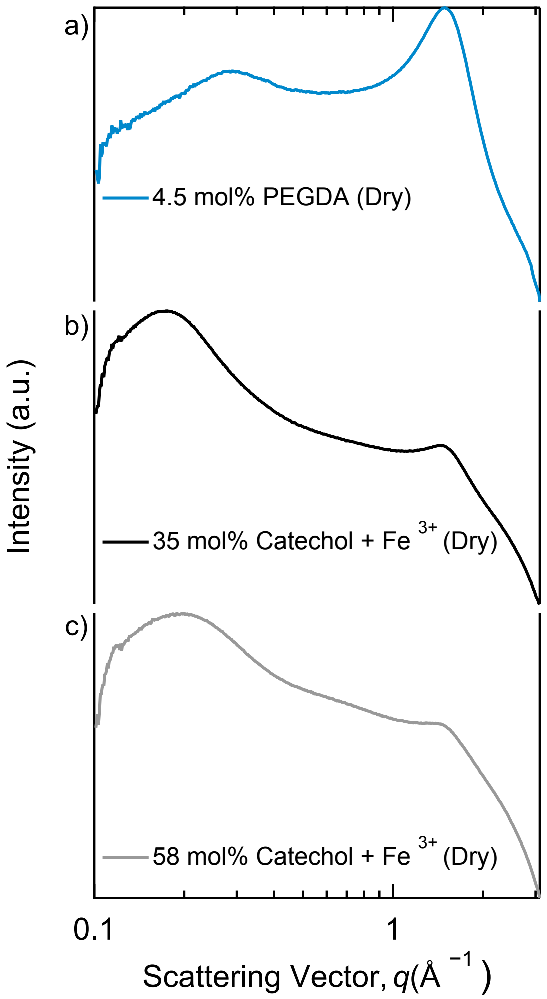

Figure S12: Wide angle x-ray scattering of dry polymer networks with comparisons between control (a) 4.5/95.5 mol% PEGDA/PEGMEA network, and catechol networks after substitution with dopamine and subsequent Fe^{3+} complexation. (b) Scattering of a 4/61/35% PEGDA/PEGMEA/catechol network, and (c) scattering of 2.9/39/58 mol% PEGDA/PEGMEA/catechol network.

References:

(1) Moon, J. D.; Sujanani, R.; Geng, Z.; Freeman, B. D.; Segalman, R. A.; Hawker, C. J. Versatile Synthetic Platform for Polymer Membrane Libraries Using Functional Networks. *Macromolecules* **2021**, *54* (2), 866–873. https://doi.org/10.1021/acs.macromol.0c02414.

(2) Yin, Q.; Wang, L.; Jiang, J.; Dai, C.; Weng, G.; He, J. Three-Dimensional Shape Transformation of Eu3+-Containing Polymer Films through Modulating Dynamic Eu3+-Iminodiacetate Coordination. *Chem. Mater.* **2022**, *34* (5), 2176–2186. https://doi.org/10.1021/acs.chemmater.1c03722.

(3) Jones, S. D.; Schauser, N. S.; Fredrickson, G. H.; Segalman, R. A. The Role of Polymer-Ion Interaction Strength on the Viscoelasticity and Conductivity of Solvent-Free Polymer Electrolytes. *Macromolecules* **2020**, *53* (23), 10574–10581. https://doi.org/10.1021/acs.macromol.0c02233.

(4) Wuttke, J. Laplace–Fourier Transform of the Stretched Exponential Function: Analytic Error Bounds, Double Exponential Transform, and Open-Source Implementation "Libkww." *Algorithms* **2012**, *5* (4), 604–628. https://doi.org/10.3390/a5040604.

(5) Love, A. *A Treatise on the Mathematical Theory of Elasticity*; 1892; Vol. 1.

(6) Ilavsky, J. Nika: Software for Two-Dimensional Data Reduction. *J. Appl. Crystallogr.* **2012**, *45* (2), 324–328. https://doi.org/10.1107/S0021889812004037.
